# Chemically Functionalized 2D Transition Metal Dichalcogenides for Sensors

**DOI:** 10.3390/s24061817

**Published:** 2024-03-12

**Authors:** Selene Acosta, Mildred Quintana

**Affiliations:** 1 Centro de Investigación en Ciencias de la Salud y Biomedicina, Universidad Autónoma de San Luis Potosí, Av. Sierra Leona 550, Lomas de San Luis, San Luis Potosí 78210, Mexico; mildred.quintana@uaslp.mx; 2 Facultad de Ciencias, Universidad Autónoma de San Luis Potosí, Av. Parque Chapultepec 1570, Privadas del Pedregal, San Luis Potosí 78295, Mexico

**Keywords:** chemical sensors, 2D transition metal dichalcogenides, chemical functionalization, biosensors, gas sensors, metal sensors, Raman sensors

## Abstract

The goal of the sensor industry is to develop innovative, energy-efficient, and reliable devices to detect molecules relevant to economically important sectors such as clinical diagnoses, environmental monitoring, food safety, and wearables. The current demand for portable, fast, sensitive, and high-throughput platforms to detect a plethora of new analytes is continuously increasing. The 2D transition metal dichalcogenides (2D-TMDs) are excellent candidates to fully meet the stringent demands in the sensor industry; 2D-TMDs properties, such as atomic thickness, large surface area, and tailored electrical conductivity, match those descriptions of active sensor materials. However, the detection capability of 2D-TMDs is limited by their intrinsic tendency to aggregate and settle, which reduces the surface area available for detection, in addition to the weak interactions that pristine 2D-TMDs normally exhibit with analytes. Chemical functionalization has been proposed as a consensus solution to these limitations. Tailored surface modification of 2D-TMDs, either by covalent functionalization, non-covalent functionalization, or a mixture of both, allows for improved specificity of the surface–analyte interaction while reducing van der Waals forces between 2D-TMDs avoiding agglomeration and precipitation. From this perspective, we review the recent advances in improving the detection of biomolecules, heavy metals, and gases using chemically functionalized 2D-TMDs. Covalent and non-covalent functionalized 2D-TMDs are commonly used for the detection of biomolecules and metals, while 2D-TMDs functionalized with metal nanoparticles are used for gas and Raman sensors. Finally, we describe the limitations and further strategies that might pave the way for miniaturized, flexible, smart, and low-cost sensing devices.

## 1. Introduction

The development of chemical sensors focuses on the design of cost-effective and reliable devices for detecting and measuring individual chemical compounds in relevant environments. Today, chemical sensors are found in every industry, for example, in food and beverage, agriculture, environmental conservation, mining, automotive, healthcare, packing, transportation, etc. [1,2,3,4]. With the increasing demand for sensors, there is a need for novel portable platforms for the detection of new analytes exhibiting high selectivity and sensitivity, low power consumption, chemical and mechanical stability, and to be accessible to all people.

The 2D materials graphene, hexagonal boron nitride (h-BN), TMDs, graphitic carbon nitrides (g-C_3_N_4_), layered metal oxides, black phosphorus (BP), and MXenes are excellent materials to fully meet these requirements [5,6,7]. The remarkable properties of 2D materials arise from electron confinement in two dimensions, the absence of strong interlayer interactions and atomic thickness, which results in a high surface area. Applicability of 2D materials in sensors depends on factors such as sheet thickness, the chemical nature of the pristine material, surface composition, and defects. Individual layers of 2D materials display significant changes in their properties compared to their bulk counterparts. For instance, the bandgap of the TMD MoS_2_ shifts from indirect to direct when a single sheet is isolated, leading to fluorescence in MoS_2_ [8]. Consequently, MoS_2_ is well-suited for optoelectronic applications, including photodetectors, electroluminescence, and luminescent probes. Extensive research has been guided using 2D materials for sensing applications due to the numerous advantages offered by their two-dimensional structure in devices [9,10,11]. The atomic thickness of 2D materials allows for the direct interaction between all atoms in the material and the analyte. Their large area not only ensures a significant active surface for sensing but also simplifies device assembly, and their unique electronic and optical properties, combined with their surface chemistry and structure, enable their interaction with specific targets, including gases, metals, and biological molecules [12,13,14,15,16].

For the implementation of 2D materials as sensors, there must be an interaction between the surface of the 2D material and the analyte, resulting in a measurable change in either the properties of the 2D material or the analyte itself. This interaction can be categorized as either covalent or non-covalent, depending on the chemical characteristics of the analyte and the 2D material. For example, graphene primarily undergoes interactions such as hydrogen–π, π-π, cation–π, and anion–π interactions due to its π electron system [17,18,19]. Interactions between 2D-TMDs and analytes are typically electrostatic and van der Waals interactions, while covalent interactions involve chemical reactions between analytes and 2D materials to form covalent bonds on their basal planes, a process known as chemical adsorption. Conversely, non-covalent interactions involve the physical adsorption of analytes onto the basal planes of 2D material sheets [5,20].

The tailored design and production of the optimal molecular interaction depend on the required type of sensor. If the sensing mechanism involves immediate response and rapid recovery, physical absorption is preferred. However, if biological analytes are to be immobilized on the surface of the 2D material, only chemical adsorption can provide the required stability. For chemical and physical adsorption, the use of pristine 2D materials in sensors often does not result in the highest sensitivities and selectivity [21,22,23]. The primary drawback is linked to the weak interaction that pristine 2D materials exhibit with analytes. For 2D-TMDs, these interactions occur as van der Waals interactions, which are indeed extremely weak forces. Dangling bonds on 2D-TMDs facilitate strong and specific interactions with analytes. Other limitations of 2D materials include agglomeration and settling in dispersion, reducing the sensing surface area. To address these disadvantages, surface modification of 2D-TMD materials has been successfully performed through defect creation or functionalization, enhancing the interaction between 2D-TMDs and specific analytes [24,25,26,27].

From this perspective, we focus on the use of TMDs as sensor platforms as they are, so far, the most studied 2D materials after graphene and graphene oxide, particularly MoS_2_. TMDs follow the chemical formula MX_2_ in their structure. M is a transition metal, and X is a chalcogen. Forty different TMDs have been reported; among them, the most studied are MoS_2_, WS_2_, WSe_2_, and MoSe_2_ [7]. A single layer of TMDs comprises three atomic stratums linked through covalent bonds, with the transition metal positioned between two chalcogens. In bulk, TMDs are layered materials whose layers are attached by van der Waals interactions. Monolayers of TMDs have astonishing properties due to the confining of charge carriers in two dimensions. These properties convert them into potential materials for chemical sensors. The d orbitals in the electronic structure of 2D-TMDs allow us to adjust their physical properties as valence electrons, carrier mobility, and chemical and mechanical stability. The 2D-TMDs have been extensively investigated for chemical sensing applications due to their high area-to-volume ratio. In most of the reported works, 2D-TMD nanosheets are obtained via chemical and liquid phase exfoliation. The 2D-TMDs obtained through chemical exfoliation processes have limitations that hinder their use in sensors; these limitations include agglomeration and settling when the nanosheets are in dispersion and a deficiency of dangling bonds to facilitate the covalent conjugation of external probes. These drawbacks can be easily tackled by means of 2D-TMDs surface functionalization, a process that can be accomplished by utilizing 2D-TMDs defects, such as chalcogen vacancies, produced during exfoliation processes.

As is widely known, the synthetic methodology used to produce 2D-TMDs influences the structural defects present in the materials. The 2D-TMDs obtained through chemical and liquid phase exfoliation tend to be rich in chalcogen vacancies [28]. The defects in 2D-TDMs dictate their surface properties and, consequently, their ability to interact with molecules. While defects may pose disadvantages in certain applications, such as optoelectronics, carefully engineered defects can introduce new and adjustable properties for applications in sensor devices. Modifying pristine 2D-TMDs to adjust their properties and improve their interaction with analytes is achieved through chemical functionalization. Functionalization involves altering the surface properties of a material by introducing or attaching chemical groups or molecules. The enhanced sensing of 2D-TMDs through functionalization has been pursued using various approaches. The 2D-TMDs are functionalized in both covalent and non-covalent protocols with molecules and biomolecules such as DNA, RNA, proteins, polymers, and nanoparticles.

Herein, we provide a summary and discussion of the recent advancements in both covalent and non-covalent functionalization of 2D-TMDs with the aim of enhancing their efficiency as chemical sensors towards different analytes. This perspective specifically focused on highlighting how surface modification of 2D-TMDs allows for tuning their properties, leading to the development of chemical sensors characterized by increased sensitivity, selectivity, and low detection limits. Biosensors, metal sensors, gas sensors, and Raman sensors are described. We further describe some strategies that might pave the way for miniaturized flexible, smart, and low-cost sensing devices. To the best of our knowledge, this is the first perspective exclusively dedicated to the chemical functionalization of 2D-TMDs for chemical sensors.

## 2. Biosensors

MoS_2_ is the most studied of the 2D-TMDs due to its physicochemical properties like large bang gap, flexibility, and photoluminescence. One of the first works of functionalized MoS_2_ used for chemical sensing was reported by Huang and collaborators in 2013 [29], where MoS_2_ sheets obtained from sonication were functionalized with Cu nanoparticles by directly performing a chemical reduction of a copper chloride onto MoS_2_ nanosheets using glucose and 1-hexadecylamine. Large area MoS_2_ nanosheets decorated with well-distributed copper nanoparticles with diameters up to 5 nm were obtained. Afterward, the synthesized material was evaluated in glucose sensing by depositing Cu-MoS_2_ in a glassy carbon electrode and using cyclic voltammetry and amperometry to seek the oxidation of glucose by Cu-MoS_2_. The Cu-MoS_2_ sensor exhibited a sensitivity of 1055 µA mM^−1^ cm^−2^ and selectivity upon ascorbic acid, uric acid, and dopamine.

**Figure 1:**
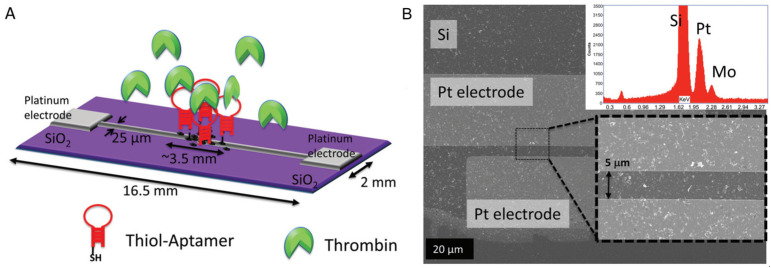
(**A**) Schematic representation of a thrombin biosensor based on aptamer functionalized MoS_2_. (**B**) SEM image of MoS_2_ deposited on a Pt electrode. The inset displays Energy Dispersive X-ray (EDAX) spectra from the zone in the square of SEM micrography. Adapted from [32].

MoS_2_ had been functionalized with different biomolecules to produce biosensors. A widely explored strategy is the functionalization of MoS_2_ with DNA. In 2014, Mei Kong et al. [30] developed a biosensor based on MoS_2_ obtained by lithium intercalation; the nanosheets exhibit a wrinkled paper-like structure. Afterward, MoS_2_ was non-covalent functionalized with a dye-labeled single-stranded DNA probe (aptamer). The DNA-MoS_2_ interaction occurs through van der Waals forces between the nucleobases and the basal plane of MoS_2_ nanosheets. In this report, the aptamer recognized the prostate-specific antigen (PSA), a biomarker used for the diagnosis of prostate cancer. The functionalization of MoS_2_ with the aptamer induced an aptamer fluorescence quenching. The sensor device was developed using the aptamer fluorescence as a transducer signal; the fluorescence of the aptamer was recuperated as it bonded with PSA and liberated from MoS_2_. This sensor had a 0.2 ng/mL detection limit and worked in human serum samples. An analogous work was reported by Zhang and collaborators in 2021 [31], where PSA was detected with a field-effect transistor biosensor device based on a DNA tetrahedron functionalized MoS_2_ followed by a functionalization with the protein biotin–anti-PSA. In this work, the authors achieved a detection limit of 1 fg/mL. This impressive result was related to the stable immobilization provided by the DNA-MoS_2_ functionalization. Following a similar strategy, Chun Lin and collaborators [32] fabricated a biosensor based on MoS_2_ functionalized with an aptamer to detect thrombin, a biomolecule used to monitor inflammation. MoS_2_ nanosheets were obtained through liquid-phase exfoliation. The sensor platform was made of SiO_2_ substrates where Pt electrodes were deposited using an e-beam cryo-evaporator, and MoS_2_ nanosheets were subsequently deposited on the electrode having sizes up to 100 nm (Figure 1B). The aptamer was immobilized on the surface of MoS_2_ by incubation at room temperature for 90 min. Their interaction was confirmed by impedance measurements, and it is based on van der Waals interactions. The detection of thrombin was made by monitoring changes in impedance resulting from the interactions between the immobilized aptamer on the electrode surface and thrombin (Figure 1A). The sensor was capable of quantifying thrombin in human serum.

**Figure 2:**
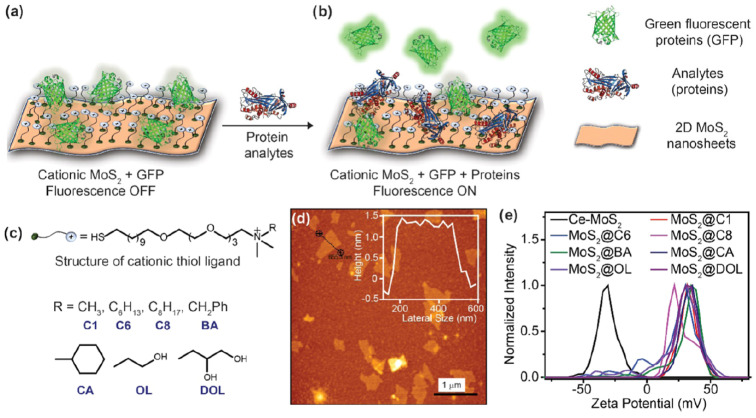
(**a**) Schematic representation of the sensor developed by Behera et al. Quenching of GFP fluorescence by cationic MoS_2_. (**b**) Addition of the analyte releases GFP from the surface of cationic MoS_2_, followed by regeneration of GFP fluorescence. (**c**) Structure of cationic thiol ligands. (**d**) Atomic force microscopy (AFM) image of chemical exfoliated MoS_2_ (Ce-MoS_2_). (**e**) ζ-Potential plot for MoS_2_ that proves thiol functionalization. Reproduced from [33].

The defects produced in MoS_2_ sheets during their synthesis can be used to assist their functionalization. Behera and collaborators [33] used the sulfur vacancies produced in MoS_2_ during exfoliation to functionalize MoS_2_ nanosheets with different cationic thiol ligands. MoS_2_ nanosheets exhibit a diameter range of 300–600 nm and a height of approximately 1.2 nm. The positive charge induced through thiol functionalization allowed the conjugation of MoS_2_ with the fluorescent protein GFP, which has a negative charge; the conjugation of GFP with functionalized MoS_2_ induced a quenching in the GFP fluorescence. Afterward, a biosensor was developed based on a displacement assay of GFP with several analytes competing for the interaction with functionalized MoS_2_. The release of GFP from functionalized MoS_2_ incited the recovery of the fluorescence (Figure 2). In this report, GFP fluorescence was used as the signal transducer to detect 15 different proteins, such as β-galactosidase and macerozyme.

**Figure 3:**
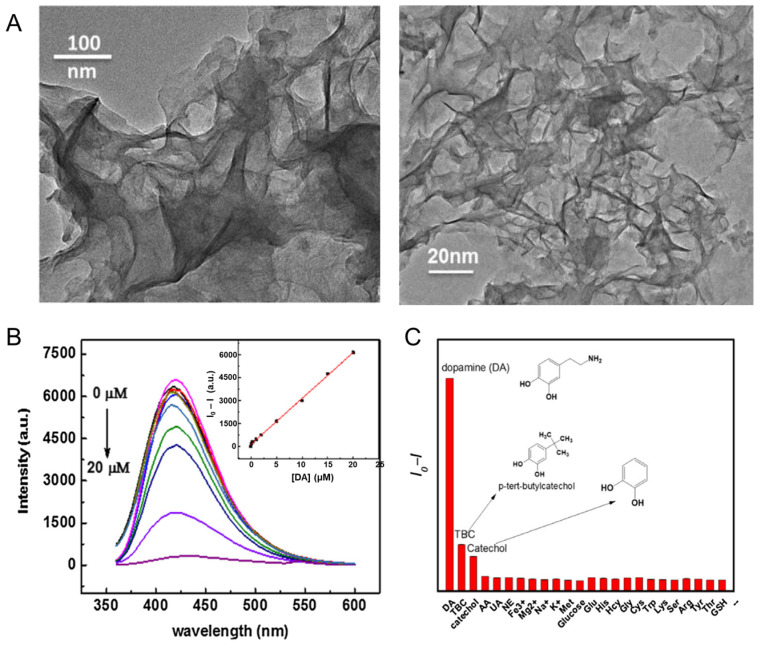
(**A**) TEM images of the TGA-MoS_2_ nanosheets. (**B**) Fluorescence emission spectra recorded for the TGA-MoS_2_ sensor at various concentrations of dopamine. Inset: standard curve established to determine the concentration of dopamine. (**C**) Fluorescence response of TGA-MoS_2_ with different guest molecules. Reproduced from [34].

The functionalization during the synthesis of MoS_2_ was explored by Xu and collaborators [34]. In this report, MoS_2_ was functionalized with thioglycolic acid (TGA) through a hydrothermal treatment where molybdenyl acetylacetonate, TGA, and sodium sulfide were the precursors, and the product of this synthesis was TGA-MoS_2_ nanosheets. The MoS_2_ nanosheets exhibit curling and overlapping due to their ultrathin characteristics and TGA surface modification (Figure 3A). The average thickness of the MoS_2_ nanosheets obtained by AFM confirmed the production of single-layer nanosheets. Additionally, TGA-MoS_2_ presented a fluorescence centered at 420 nm, with intensity decreased in the presence of dopamine (Figure 3B,C). Xu and colleagues reported a facile method where dopamine can be sensed using the fluorescence of TGA-MoS_2_ as a transducing signal, achieving a detection limit of 27 nM.

**Figure 4:**
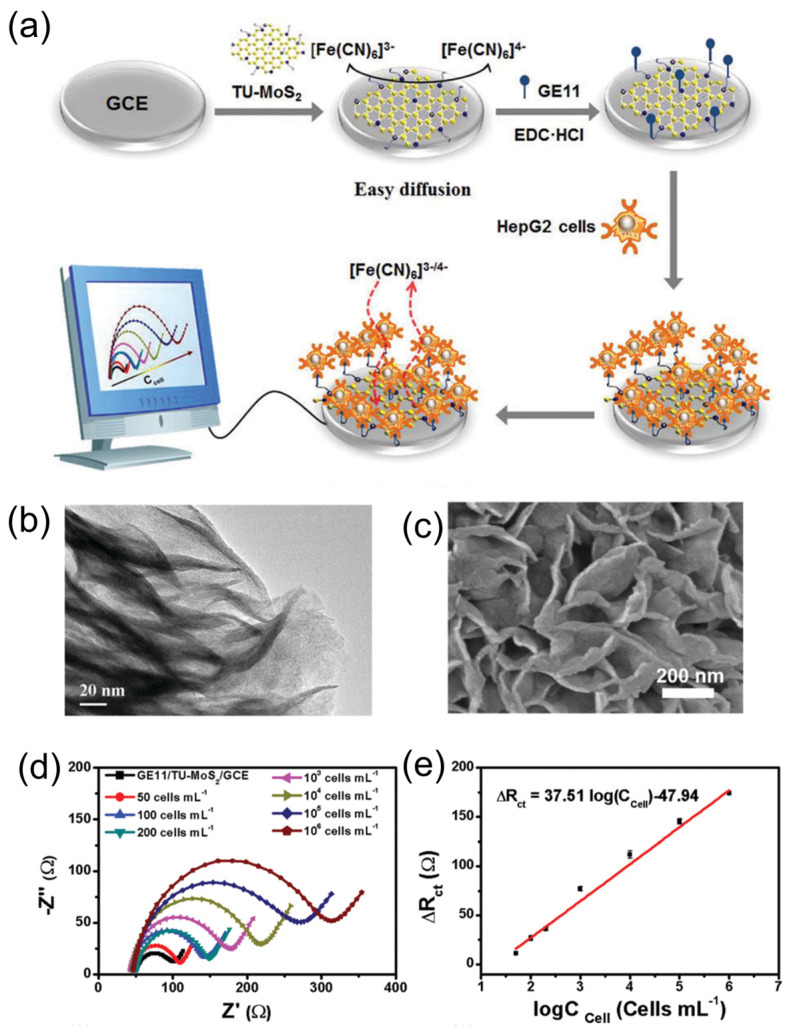
(**a**) Schematic illustration for fabrication of biosensor based on surface functionalized thiourea–MoS_2_ nanosheets. (**b**) TEM image of thiourea–MoS_2_ nanosheets. (**c**) SEM image of thiourea–MoS_2_ nanosheets. (**d**) Electrochemical impedance spectroscopy (EIS) response of GE11/TU-MoS_2_ electrode to various concentrations of HepG2 cells. (**e**) Calibration curve of ΔR_ct_ vs. logC_cell_. Adapted from [35].

Guo and collaborators [35] functionalized MoS_2_ nanosheets with thiourea via a microwave-assisted hydrothermal route. Nanosheets with lateral size range from 200 to 300 nm were reported. The change in the interlayer spacing from 0.62 nm in pristine MoS_2_ to 0.92 nm in thiourea–MoS_2_, observed in high-resolution transmission electron microscopy (HR-TEM) images, is evidence of MoS_2_ functionalization. Afterward, a biosensor was constructed using the amino group in thiourea to attach an antigen named GE11, which recognizes the EGFR receptor present in human liver cancer cells. The biosensor was developed by measuring the change in the impedance as a function of cell concentration, allowing a low detection limit of 50 cells/mL (Figure 4).

Singh and collaborators [36] functionalized MoS_2_ with cetyltrimethyl ammonium bromide (CTAB) by sonicating MoS_2_ powder in water with CTAB (1%) solution to accomplish the conjugation of an antibody that recognized the microorganism *Salmonella typhimurium* in a microfluidics electrode. Transmission electron microscopy (TEM) analysis showed the obtention of CTAB-MoS_2_ nanosheets with lateral sizes ranging from 50 to 200 nm. The observation of single MoS_2_ layers indicates that CTAB can efficiently assist in the exfoliation of MoS_2_ in water. Additionally, SEM analysis showed that CTAB-MoS_2_ are mostly arranged in a flower-like structure. *S. typhimurium* is a Gram-negative bacterium responsible for a great part of human food poisoning in the world. Singh reports on a microfluidic immunosensor utilizing electrochemical impedance spectroscopy for the detection of *S. typhimurium* with a sensitivity of 1.79 kΩ/CFU^−1^ mL cm^−2^. Zhang and collaborators [37] developed an electrochemical sensor toward the PIK3CA gene, which is associated with lung cancer. The sensor was based on MoS_2_ nanosheets functionalized with riboflavin 5′-monophosphate sodium salt (FMS); the functionalization was carried out during the synthesis of MoS_2_ through liquid-phase exfoliation. FMS-functionalized MoS_2_ has wrinkly layered structures and was successfully dispersed in water, contrary to pristine MoS_2_, in which agglomeration was observed in SEM images. To fabricate the sensor, FMS-MoS_2_ nanosheets were deposited on a glassy carbon electrode, and a ssDNA was bonded covalently to the FMS-MoS_2_ using the amine groups of the ssDNA and the phosphonate groups of the FMNs. The ssDNA was complementary to the PIK3CA gene, and cyclic voltammograms were measured when the sensor was in the presence and absence of the PIK3CA gene.

Even though most of the sensors based on MoS_2_ functionalized with metal nanoparticles had been studied for the detection of toxic and hazardous gases, these materials may also work for the detection of other kinds of molecules [38,39]. For example, Wang et al. [40] synthesized MoS_2_ functionalized with Au-nanoparticles and used it for the construction of a DNA sensor based on electrochemiluminescence. SEM characterization showed a smooth and large surface area of the produced MoS_2_ nanosheets and Au-nanoparticles with 13 nm in diameter that were well dispersed in the surface of MoS_2_. The sensor was developed in a sandwich type where Au-MoS_2_ were functionalized with DNA as well as CdS/ZnS quantum dots coated with polyethyleneimine. The QDs were attached to the DNA that was going to be detected and Au-MoS_2_ to a reporter DNA. This sensor can detect DNA at a concentration of 0.05–1000 fM. Different types of nanoparticles have been used to functionalize MoS_2_ and increase its sensing performance. In 2021, Xiao et al. [41] deposited MoS_2_ nanosheets in a screen-printed electrode and grew Au-Pt nanoparticles onto the MoS_2_ surface using electrodeposition. The morphology of the electrode was characterized using SEM, and a clear difference was observed in the electrode with and without Au-Pt MoS_2_ functionalization. The bare electrode was smoother than the functionalized one. Au-Pt nanoparticles had a diameter ranging from 110 to 130 nm. The fabricated sensor was used to detect lactic acid, a metabolite used as a biomarker in medical diagnosis. Au-Pt-NPs functionalization increased the electron transfer rate and worked as an oxidant of lactic acid in the sensing reaction.

**Figure 5:**
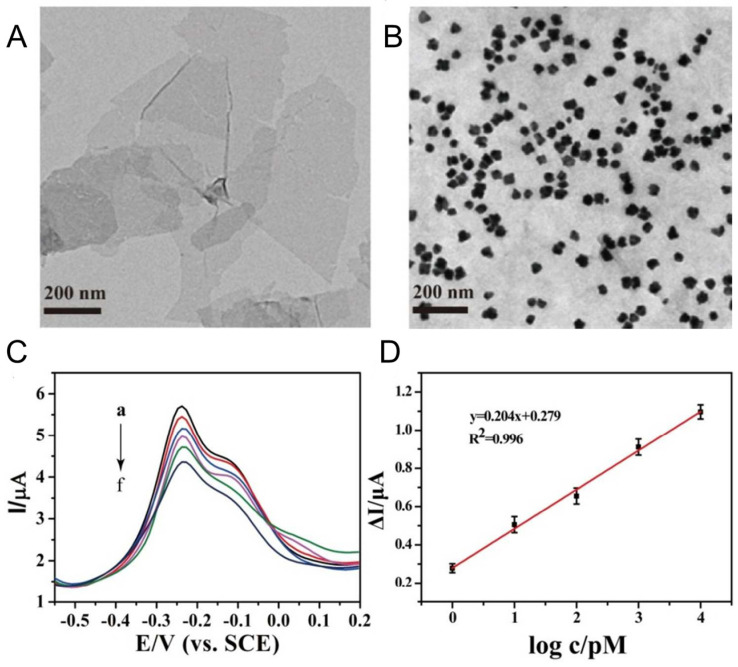
(**A**) Transmission electron microscopy of WS_2_ and (**B**) MoS_2_-Thi-AuNPs nanocomposite. (**C**) MoS_2_-Thi-AuNPs response to numerous miR-21 concentrations (a–f: 0, 1.0, 10, 100, 1000 and 10,000 pM). (**D**) Calibration curves of sensor responses to miR-21. Adapted from [42].

The co-functionalization of MoS_2_ nanosheets with a thiol end molecule and metal nanoparticles for the development of an RNA biosensor was explored by Zhu and collaborators [42]. In this report, the synthesis of the MoS_2_-Thi-AuNPs nanocomposite was achieved through a microwave-assisted hydrothermal method, followed by assembly onto a glassy carbon electrode. TEM characterization showed the few layers of exfoliation of MoS_2_ and the formation of Au-nanoparticles on their surface with an average diameter of 40 nm. DNA conjugation was used as a recognition probe, and thionine acted as an electrochemical indicator for the detection of RNA and as a reducing agent for the formation of Au-nanoparticles (Figure 5). This sensor device was able to detect RNA with a limit detection of 0.26 pM and the possibility of detecting specific RNA in human serum.

Emerging viruses, such as COVID-19, responsible for the pandemic that emerged in 2019 in Wuhan, China, highlighted the importance of the fast and simple development of innovative biosensors. The 2D materials properties allowed the fabrication of COVID-19 biosensors with high sensitivity [43]. Peng and collaborators [44] used carboxyl functionalized MoS_2_ deposited on ITO to identify SARS-CoV-2 and its S protein by n-IR plasmonic response at 1550 nm. The linear detection range for the SARS-CoV-2 ranged from 0 to 67.87 nM, and its S glycoprotein was between 0 and 301.61 nM.

**Figure 6:**
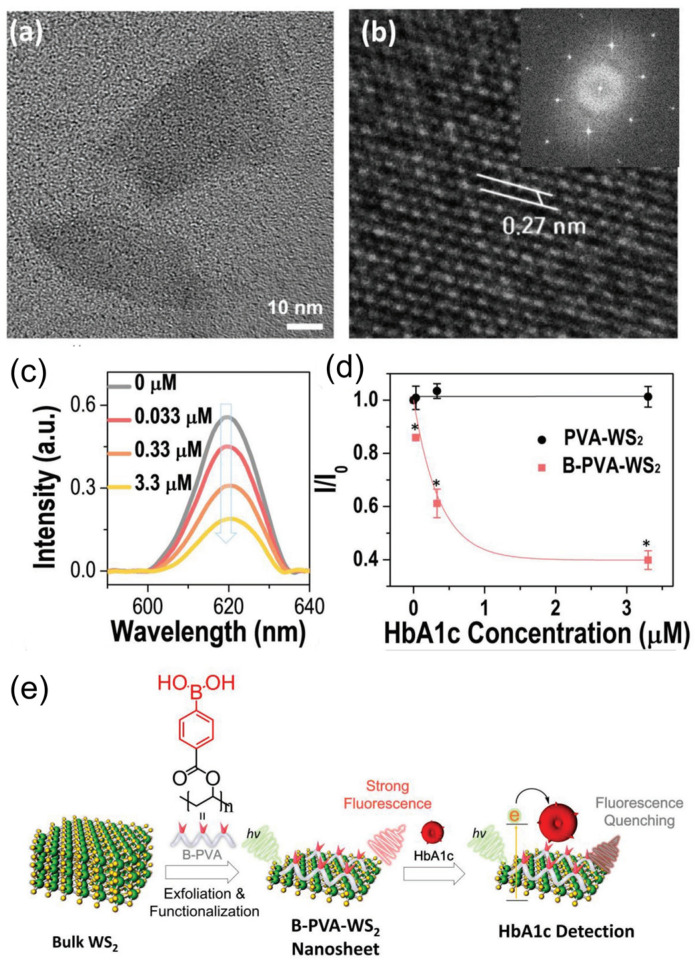
(**a**) TEM of B-PVA-WS_2_ nanosheets. (**b**) HRTEM image of B-PVA-WS_2_. (**c**) Photoluminescence spectra of B-PVA-WS_2_ at various concentrations of glycated hemoglobin. (**d**) Assessment of photoluminescence quenching responses between PVA-WS_2_ (non boronic acid present) and B-PVA-WS_2_ in the presence of glycated hemoglobin. * *p* < 0.001 versus negative control. (**e**) Schematic diagram of biosensor developed by Yang and collaborators. Adapted from [46].

The functionalization of other 2D-TMDs and their application in different biosensors has also been investigated. The functionalization of WS_2_ with the porphyrin hemin was reported by Chen and collaborators [45]. The functionalization was confirmed by UV-vis absorption spectra and X-ray photoelectron spectroscopy (XPS); SEM characterization showed the layered structure of hemin–WS_2_, indicating that functionalizing WS_2_ with hemin does not affect its prime structure. The interaction between WS_2_ and hemin was carried out through van der Waals interactions. The hemin–WS_2_ nanosheets presented peroxidase-like activity, and the oxidation of a substrate produced a colored reaction. Chen and colleagues used this colored reaction to develop a simple glucose sensor using glucose oxidase and the hemin-functionalized WS_2_. The glucose sensor reported in this work had a detection limit of 1.5 × 10^−6^ mol L^−1^. This sensor can be considered low-cost production as it is based on a colorimetric reaction easily detected by the naked eye. Yang et al. [46] functionalized WS_2_ nanosheets with a modified polymer to develop a biosensor of glycated hemoglobin. The polymer used was boronic acid-modified polyvinyl alcohol (B-PVA), and WS_2_ nanosheets were functionalized with it during their liquid-phase exfoliation. TEM images of the B-PVA-WS_2_ nanosheets revealed a clearly defined thin 2D structure featuring a lateral size of approximately 50 nm. The electron diffraction pattern of the B-PVA-WS_2_ demonstrates their inherent 2H phase following exfoliation and functionalization with B-PVA. B-PVA-WS_2_ presented intense fluorescence when excited at 532 nm, and its fluorescence was quenched in the presence of glycated hemoglobin. The sensor was developed using the B-PVA-WS_2_ fluorescence intensity and detected glycated hemoglobin down to the concentration 3.3 × 10^−8^ M (Figure 6).

Luo et al. [47] reported MoSe_2_ nanosheets that were synthesized hydrothermally and treated with Ar plasma to induce selenium vacancies. Additionally, nitrogen atoms were introduced to the vacancies through N_2_ plasma. The morphological change of MoSe_2_ during plasma functionalization was monitored through SEM. Prior to functionalization, MoSe_2_ presented a smooth layered structure with an average lateral size of 60 nm; after plasma treatment, the surface of MoSe_2_ was rough and there was a mutilation of the edges. The presence of Nitrogen functionalization and Se vacancies were observed by HRTEM and EDS analysis. Afterward, N-MoSe_2_ nanosheets were deposited on a glassy carbon electrode to evaluate its sensing performance towards H_2_O_2_. The sensor developed by Luo demonstrated effective performance in detecting hydrogen peroxide, boasting a low detection limit of 12.6 nmol/L. Finally, in a recent work reported by Song et al. [48], fluorinated WSe_2_ nanosheets (F-WSe_2_) were used to develop an efficient platform for detecting cytosolic miRNA. The morphological properties of fluorinated WSe_2_ nanosheets were characterized through TEM and AFM. F-WSe_2_ had an average particle size of 120 nm and an average thickness of 1.1 nm; this was evidence of the obtention of single-layer F-Wse_2_. To fabricate a sensor device, a fluorophore-labeled single-stranded DNA (ssDNA) was adhered to the F-WSe_2_ nanosheet surface through electrostatic interaction and π-π stacking. This bonding resulted in a significant quenching of the ssDNA fluorescence. The formation of double-stranded complexes occurs as single-stranded DNA (ssDNA) hybridizes with its target in the cytosol. Consequently, the target strand was released from the surface of F-WSe_2_, allowing the retention of probe fluorescence. Successful detection of intracellular miRNA-21 and miRNA-210 was achieved with this novel sensor. In this work, it was reported for the first time the cytosolic delivery of 2D nanomaterials.

## 3. Metal Sensors

The sensing of extremely low concentrations of heavy metals in water is crucial for environmental care. In 2018, Gan and collaborators [49] searched for an MoS_2_ functionalization that allowed for the change in the electronic surface of MoS_2_ nanosheets without changing their original lattice structure. To achieve this, MoS_2_ was exfoliated with N, N-dimethylformamide (DMF), 1-methyl-2-pyrrolidinone, and formamide, expecting to induce nitrogen functionalities on the MoS_2_ nanosheets. AFM analysis indicated that MoS_2_ was obtained as nine-, five-, and eleven-layered materials depending on the solvent used. In addition, TEM analysis showed that MoS_2_ exfoliated with formamide results in the smaller MoS_2_ nanosheets obtained. Through DFT calculations, Gan and collaborators probed that Mo-N covalent bonds can be formed between MoS_2_ nanosheets and the nitrogen atoms present in the solvent molecules. The N-MoS_2_ functionalized surfaces were then deposited on a glass carbon electrode and used to detect Cd^2+^ from water with a detection limit of 0.2 nM. In the same research direction, Bazylewski et al. [50] developed a Cd^2+^ sensor based on L-cysteine functionalized MoS_2_. First, MoS_2_ nanosheets were obtained from ultrasonication in a mix of thioglycolic acid and water to obtain carboxylated MoS_2_ (COOH-MoS_2_). Afterward, the carboxyl group was used in an amide cross-linking reaction to attach L-cysteine to MoS_2_ nanosheets. Thin films of Cys-MoS_2_ were assembled by vacuum filtration using poly(ether)sulfone as support. SEM analysis of the obtained films showed that MoS_2_ nanosheets tend to form clusters with a thickness ranging from 100 nm to 2 μm. More uniform films were observed in the presence of L-cysteine as a result of cross-linking induced by cysteine. The Cys-MoS_2_ films were integrated into a chemiresistor whose resistivity increased in contact with water containing 5 ppb of Cd^2+^. The device developed by Bazylewski and collaborators operated in a range of 1–500 ppb and was selective for Cd^2+^ detection.

**Figure 7:**
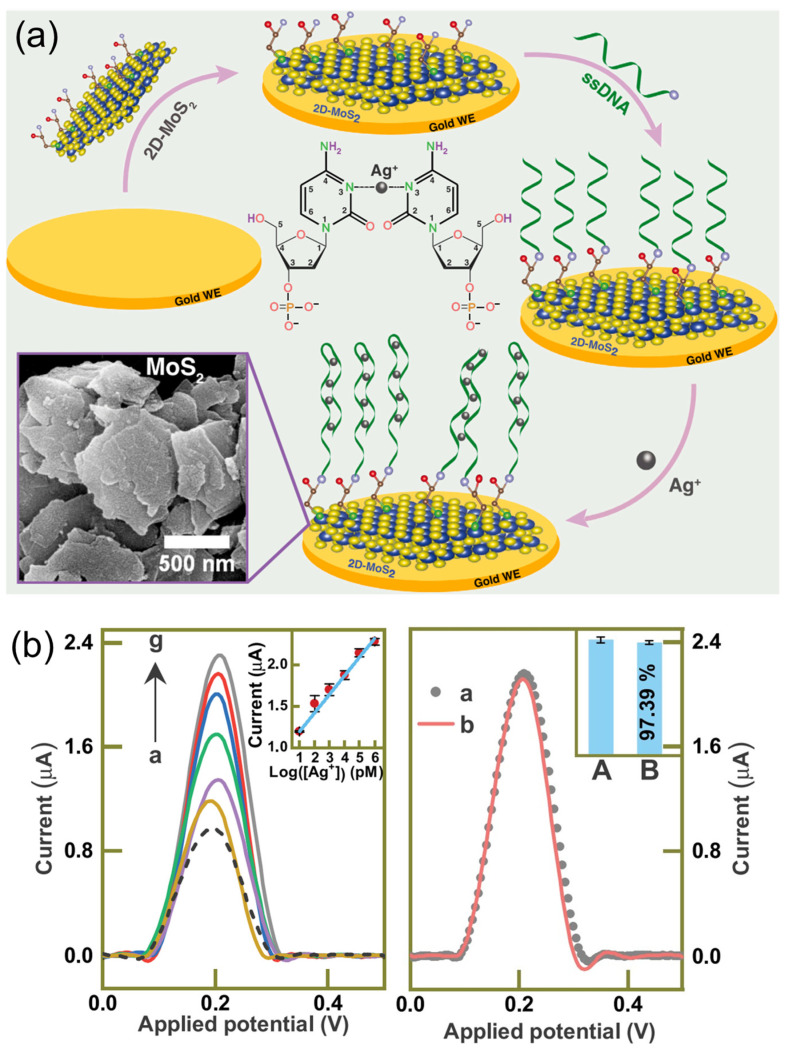
(**a**) Schematic representation of the Ag^+^ sensor reported by Pal and collaborators. The inset shows the FESEM image of COOH-MoS_2_ nanosheets. (**b**) **Left**. Square wave voltammetry (SWV) response of the sensor developed by Pal and colleagues at various concentrations of Ag^+^ (Blank sample, 10 pM, 100 pM, 1 nM, 10 nM, 100 nM, 1 μM) (calibration curve as inset). **Right**. Re-usability performance (a, after; b, before) of the Ag^+^ sensor. The inset shows reusability after 6 months. Adapted from [51].

The sensing of silver ions had been performed using a biosensor based on functionalized MoS_2_. Pal and collaborators [51] functionalized MoS_2_ with carboxyl groups by sonication with potassium hydroxide and monochloroacetic acid in deionized water. FESEM was used to analyze the layered structure of functionalized MoS_2_ and Raman spectroscopy to prove the COOH-functionalization of MoS_2_. XRD showed that after functionalization, MoS_2_ nanosheets conserve their regular hexagonal 2H polycrystalline crystal structure. Afterward, the carboxyl–MoS_2_ nanosheets were attached to a gold working electrode and functionalized with a poly(cytosine) oligonucleotide comprising 20 bases. The sensing of Ag^+^ ions was achieved by exploiting the conjugation between cytosine and Ag^+^ (cytosine-Ag(I)-cytosine). The sensing was carried out by dipping the fabricated electrode in Ag^+^-containing solutions and using the square wave voltammetry (SWV) method (Figure 7). The Ag^+^ sensor device reported by Pal et al. had a limit of detection of 0.8 pM in potable water, which makes it a potential candidate for water remediation applications.

**Figure 8:**
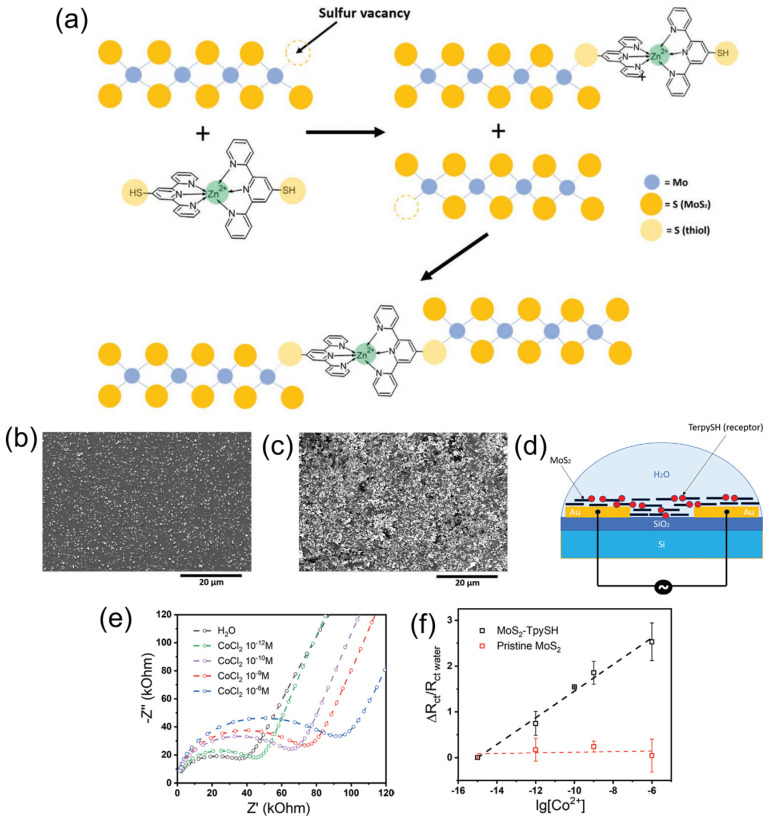
(**a**) Schematic representation of the functionalization of MoS_2_ by repairing the sulfur vacancies with thiolate molecules. (**b**) SEM image of film made of pristine MoS_2_. (**c**) SEM image of film made of thiol functionalized MoS_2_. (**d**) Schematic representation of the fabricated sensor. (**e**) Electrochemical impedance spectroscopy (EIS) sensor response at various concentrations of Co^2+^. (**f**) Calibration curves of the sensors comparing pristine and functionalized MoS_2_. Adapted from [52].

Recently, in work reported by Zhuravlova et al. [52], the sulfur vacancies present on MoS_2_ obtained by liquid-phase exfoliation were used to facilitate its functionalization with a specific receptor for Co^2+^, having a thiol termination (2,2′:6′,2″-terpyridine-4′-thiol). The modified sheets were deposited on SiO_2_ to obtain films and then placed in a gold electrode system to develop an electrochemical Co^2+^ sensor. The structural properties of the functionalized MoS_2_ film were obtained by means of SEM, XPS, and Raman spectroscopy. The thiol-functionalized MoS_2_ film had an improved coverage compared to the film made of pristine MoS_2_. Hence, the thiol functionalization worked as a cross-linker among MoS_2_ adjacent layers. XPS analysis showed a decrease in the vacancy defects of thiol-functionalized MoS_2_; this was evidence for the covalent functionalization of MoS_2_ conducted on the sulfur vacancies. Raman spectra showed that the functionalization process does not destroy or alter the MoS_2_ sheets. The sensor device was able to detect Co^2+^ from water with a limit detection of 1 ppm and was able to be selective towards Co^2+^ in the presence of K^+^, Ca^2+^, Mn^2+^, Cu^2+^, Cr^3+^, and Fe^3+^ (Figure 8). This work showed the facile functionalization of defective MoS_2_ using the MoS_2_ sulfur vacancies and a thiol end in the functional molecule. These results suggested that specific sensors can be created for detecting different heavy metals by just changing the receptor chemical properties.

**Figure 9:**
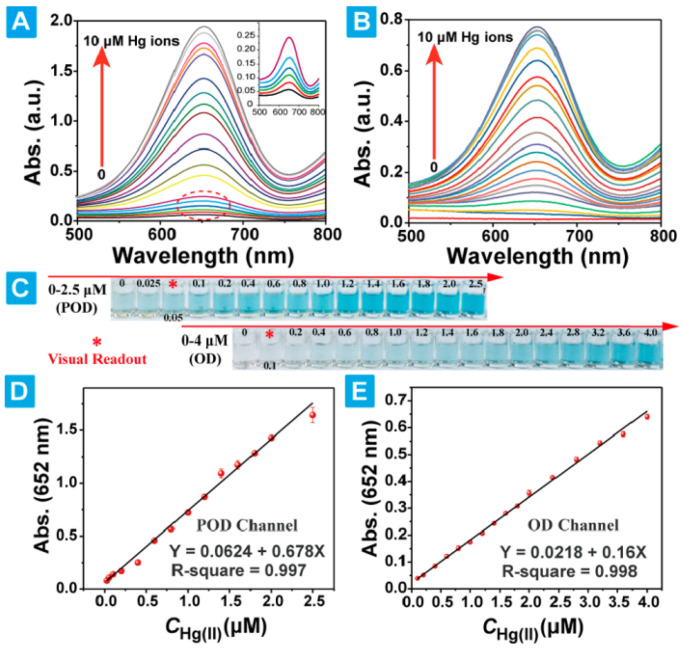
Colorimetric Hg^2+^ assay using CS-MoSe_2_ nanosheets. (**A**,**B**) UV absorption spectra recorded for reaction systems containing Hg^2+^, CS-MoSe_2_ NS, H_2_O_2_, and TMB at different concentrations. (**C**) Color changes in the reaction systems correlated with Hg^2+^ concentration. (**D**,**E**) Linear relationships between Hg^2+^ concentration and absorbance established based on the UV absorption spectra. Adapted from [53].

Huang and collaborators [53] reported a sensor based on chitosan-functionalized MoSe_2_ nanosheets to detect Hg^2+^ in water. The functionalized material was acquired through a single-step ionic liquid-assisted grinding method involving simultaneous exfoliation and functionalization. TEM and AFM analysis of the functionalized material demonstrated the obtention of mostly MoSe_2_ single layers and a rouge surface due to chitosan coating. The interaction between MoSe_2_ and chitosan was monitored using FTIR spectroscopy. The sensing mechanism was based on the capability of Hg^2+^ ions to reduce chitosan using 3,3′,5,5′-tetramethylbenzidine as a colorimetric indicator. The calibration curves were generated with the absorbance spectra; the limit of detection of this sensor was 3.5 nM Hg^2+^ (Figure 9). To validate the selectivity toward Hg^2+^ of the developed sensor, several cations and anions were tested in the colorimetric reaction in the presence and absence of Hg^2+^; the Hg^2+^ sample showed the highest absorbance at 652 nm and deep blue color compared with the other ions. Hence, the sensor developed by Huang et al. is selective and stable.

The tuning of chemical and mechanical surface characteristics in MoS_2_ thin films through the application of diazonium chemistry was performed by Saha and colleagues in 2022 [54]. Aryl diazonium chemistry involving both electron-donating (4-tertbutyl) and electron-withdrawing (4-nitro) substituted groups was used to modify peroxide exfoliated MoS_2_ films. SEM analysis of non-functionalized MoS_2_ film revealed uniformly distributed multilayered sheets. In contrast, the functionalized MoS_2_ films maintained a similar morphology, although some irregular features were observed, likely resulting from chemical surface modification. In this study, the degree of chemical interaction between distinct metal ions (Fe^2+^, Zn^2+^, Cu^2+^, and Co^2+^) and untreated and modified MoS_2_ films was investigated. Untreated films were susceptible to interaction with specific metal ions, whereas the surfaces of the modified films were observed to be entirely passivated. These results indicate that the surface functionalization of TMDs with specific molecules, such as diazonium salts, can be an outstanding option to tune the selectivity of TMDs for the detection of specific metal ions.

## 4. Gas Sensors

Atmospheric pollution is one of the biggest problems in environmental care. The development of highly sensitive and selective toxic gas sensors is an important goal to be achieved. In this direction, 2D-TMDs are promising materials to be used in gas sensors due to their high surface-to-volume ratio. The functionalization of 2D-TMDs with metal nanoparticles has been widely investigated because this type of functionalization has several effects on the sensing performance of these materials. Nanoparticles can exhibit the capability to alter the predominant type of charge carriers in TMDs, leading to distinct responses to molecules. Furthermore, nanoparticles increase the surface area of sensors, facilitating analyte diffusion, and they can also boost electron transfer between the sensor and analytes. Moreover, nanoparticles can also play a catalytic role in improving the dissociation and diffusion of analytes. Cho et al. [55] developed a volatile organic compound sensor based on MoS_2_ functionalized with Au nanoparticles. First, MoS_2_ nanosheets were obtained from liquid-phase exfoliation by sonicating MoS_2_ powder. Then, Au nanoparticles were grown onto MoS_2_ sheets. Monolayers and a few layers of MoS_2_ were observed by TEM. Au nanoparticles grown on MoS_2_ nanosheets had a diameter size lower than 10 nm. An Au-MoS_2_ thin film was fabricated using vacuum filtration and located on the surface of a µ-electrode-printed substrate. The change in the resistance of Au-MoS_2_ was measured while exposed to 100 ppm of VOC analytes such as toluene, hexane, ethanol, and acetone. The Au-MoS_2_ sensor, after exposure to the analytes toluene and hexane, did not show an important change in the resistance. Conversely, the sensor showed the best response to the sensing of acetone. The most interesting feature of this report relies on the observed change of response of Au-MoS_2_ to different VOCs depending on the sensing molecule. This feature was related to the change of the MoS_2_ charge transfer type from p-type in pristine MoS_2_ to n-type in Au-MoS_2_.

**Figure 10:**
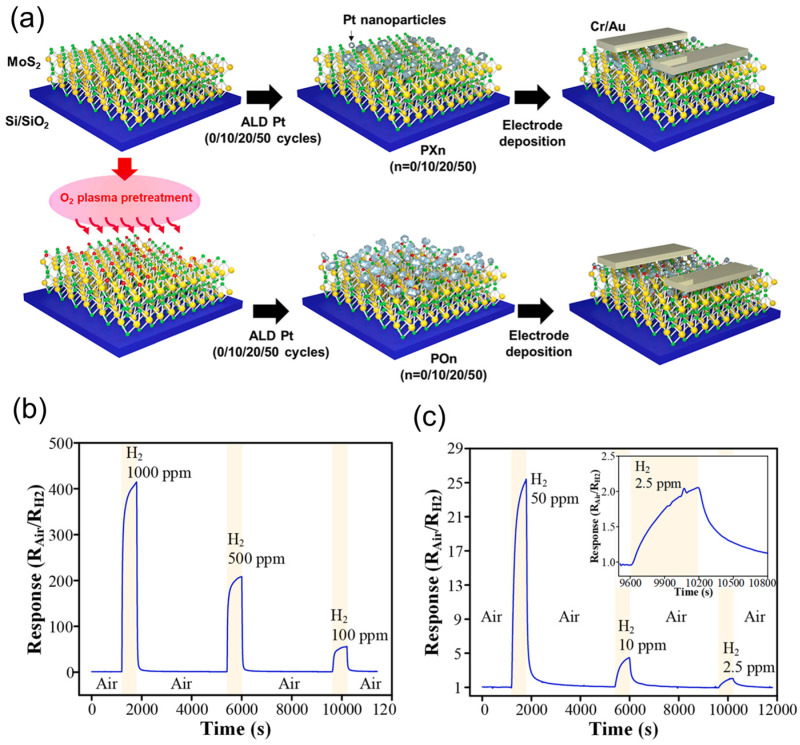
(**a**) Schematic representation of the fabrication process for the Pt-MoS_2_ H_2_ sensor. (**b**) Response of the Pt-MoS_2_ sensor on exposure to hydrogen at various concentrations from 1000 ppm to 100 ppm. (**c**) Response of the Pt-MoS_2_ H_2_ sensor on exposure to hydrogen at various concentrations from 50 ppm to 2.5 ppm. Adapted from [58].

Likewise, Bhardwaj and collaborators [56] functionalized MoS_2_ with noble metal nanoparticles to be used as active materials in the detection of VOCs. In this work, MoS_2_ nanosheets were grown directly on cellulose using the hydrothermal method. Au, Pd, and Pt were deposited on MoS_2_ using a spray coating method. MoS_2_ showed a micro-flower morphology, whereas Au nanoparticles with an average diameter size of 12 nm were observed to cover completely the surface of MoS_2_. Pd and Pt nanoparticles had average diameters of 54 nm and 77 nm, respectively. Pd and Pt nanoparticles had a wide separation in MoS_2_ nanosheets. The gas sensor performance of the functionalized MoS_2_ was carried out at 50 °C with seven different VOCs at various ppm concentrations. The nanoparticle functionalized sensors showed an increment in the sensing response compared to pristine MoS_2_, and Au-MoS_2_ showed a better response to the lowest concentration of acetone. The use of cellulose as substrate provided stable baseline resistances and less sensitivity to humidity. Chacko et al. [57] reported a gas sensor based on MoS_2_ functionalized with metals, specifically nickel and palladium. The metal functionalization was carried out by adding the metal precursors during the hydrothermal synthesis of MoS_2_ to obtain MoS_2_-Ni and MoS_2_-Pd. The MoS_2_ nanosheets exhibit a curly-like morphology of assembled nanosheets in FESEM analysis; this arrangement indicates a high surface area, which is important for good sensing performance. The metal–MoS_2_ nanosheets were deposited on silicon wafer sensing devices and tested for different toxic and hazardous gases such as NH_3_, H_2_S, NO, and NO_2_. It was observed that Ni-MoS_2_ sensors demonstrated heightened sensitivity in detecting H_2_S gas. Meanwhile, the Pd-MoS_2_ sensor displayed exceptional sensitivity, stability, and notable selectivity in detecting NO. These high sensitivities are linked to the interaction between the metals with gas molecules and the synergy with the high surface area of MoS_2_ sheets. In the same direction, Lee and colleagues [58] reported in 2022 the functionalization of MoS_2_ nanosheets with Pt-nanoparticles to improve its gas sensing performance toward H_2_. In this work, CVD-grown MoS_2_ was first functionalized with oxygen using O_2_ plasma functionalization; then, Pt nanoparticles were grown through atomic layer deposition. Through AFM analysis, it was determined the obtention of ~eight-layer MoS_2_ and that the oxidation process did not induce damage in the MoS_2_ structure. The Pt nanoparticles were grown more homogenously on the surface of oxygen-functionalized MoS_2_, and they showed an island type grown in pristine MoS_2_. The oxygen groups worked as nucleation sites for the formation of Pt nanoparticles homogeneously on the MoS_2_ surface. The sensor device was fabricated by deposition of Cr/Au electrodes on the Pt-functionalized MoS_2_ using e-beam evaporation (Figure 10). The sensor presented by Lee exhibited a reduction in its resistance when exposed to H_2_. The sensor demonstrated a significant relative resistance change, exceeding 400 times the initial resistance, with a detection limit for H_2_ set at 2.5 ppm.

**Figure 11:**
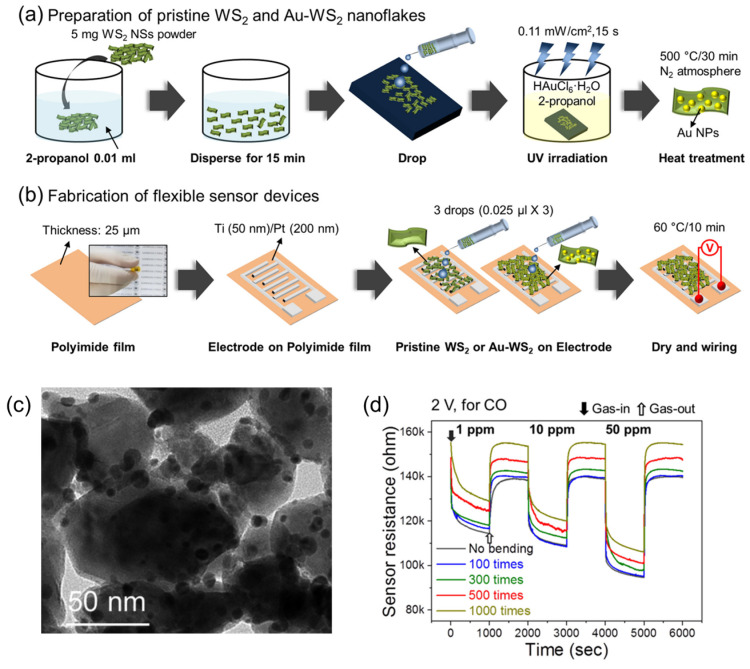
(**a**) Schematic illustration of the preparation procedures for unmodified and gold-functionalized WS_2_ nanoflakes. (**b**) Construction of flexible gas sensors developed by Kim et al. (**c**) TEM image of Au-WS_2_ nanoflakes. (**d**) Resistance curves of the Au-functionalized WS_2_ gas sensor analyzed under two conditions: without bending and with bending at a radius of curvature of 4 mm, varying the number of bending cycles. Adapted from [61].

Other metal particle functionalized 2D-TMDs have been evaluated as gas sensors. The doping of WSe_2_ with noble metals such as Pd, Ag, Au, and Pt was evaluated as a versatile approach to improve toxic gas sensing of CO_2_, NO_2_, and SO_2_. Adsorption energy, band structure, and charge transfer were computed using first-principles density functional theory, finding that NO_2_ adsorption on Ag-WSe_2_ was the most energetically stable configuration, advising the development of an efficient NO_2_ gas sensor [59]. The functionalization of WS_2_ with Ag nanowires to improve their sensing performance towards NO_2_ and acetone was reported by Yong Ko et al. [60]. Large layers (4 inches) of WS_2_ were obtained by atomic layer deposition on an 8-inch SiO_2_ wafer; with this technique, the number of WS_2_ layers can be controlled, and one-, two- and four-layer WS_2_ films were obtained. Then, Ag nanowires (AgNWs) were deposited on the WS_2_ layer using spin coating. AgNWs coverage was found to be 2.5% of the total area of the WS_2_ nanosheet. Finally, for the sensor device fabrication, Cr/Au electrodes were deposited on the surface of WS_2_. The sensing performance of four-layer WS_2_ was better for the detection of acetone and NO_2_ compared to one-layer WS_2_, which has an unobservable response. The authors compared the sensing performance of pristine and modified WS_2_, observing that functionalized AgNWs-four-layer-WS_2_ increases by 667% its response to NO_2_ molecules. On the other hand, Kim and collaborators [61] reported a sensor based on WS_2_ functionalized with gold nanoparticles. Au nanoparticles were grown by photoreduction on WS_2_ layers and deposited on polyamide as a substrate. Au nanoparticles with diameters of ~7.4 nm were obtained on the surface of WS_2_ when irradiated 15 s with UV light. The sensors were reported to be flexible with high stability and present a good selectivity towards CO gas molecules with a sensor response of 1.48 (ratio of resistance in air and resistance in the presence of 50 ppm CO) (Figure 11). Likewise, Zhang and collaborators [62] developed a CO sensor based on Pd nanoparticles functionalized WSe_2_. Films of Pt-WSe_2_ were prepared using the hydrothermal method. From SEM analysis, it can be observed that WSe_2_ exhibits hexagonal nanosheet morphology and, in contrast to pristine WSe_2_, the Pd-WSe_2_ composite displays a surface with increased roughness. In addition, the Pd particles are clustered in the form of small spheres on the WSe_2_ surface. The dispersion of Pd-WSe_2_ was applied to a sensor device through spray-coating, with Al_2_O_3_ serving as the substrate and Pt as the electrode. The Pd-WSe_2_ thin film sensor exhibited outstanding sensing capabilities for CO gas molecules with a relative response of 15% and a detection limit of 1 ppm. Similarly, Sakhuja et al. [63] functionalized WSe_2_ with metal nanoparticles to evaluate its performance as a gas sensor. In this case, Au and Pt nanoparticles were grown on the WSe_2_ surface by metal salts reduction. TEM analysis showed the layers of WSe_2_ covered with small Au/Pt nanoparticles. As expected, the functionalization of WSe_2_ improved its performance at sensing NO_2_ molecules. Au-WSe_2_ showed a response to NO_2_ of 170% and a detection limit of 100 ppb.

Most of the functionalization performed on MoS_2_ for gas sensor applications implies its decoration with metal nanoparticles. However, other kinds of functionalization have also been tested. Kim and colleagues [64] fabricated a VOCs sensor based on MoS_2_ functionalized with mercaptoundecanoic acid. The functionalization was corroborated by XPS and FTIR analysis and was performed over the surface defects on MoS_2_. The sensor developed by Kim et al. presented high sensitivity towards VOCs (toluene, hexane, ethanol, propanal, and acetone) with responses up to 15% for acetone and limiting concentrations down to 1 ppm. The advantage of functionalizing with thiol ligands instead of metal nanoparticles lies in the simplicity and high reproducibility of carrying out the thiol ligand functionalization.

Humidity sensors based on functionalized 2D-TDMs have been explored. In 2020, Gupta et al. [65] used a wet chemical method to generate WS_2_ functionalized with Pt nanoparticles for the development of a humidity sensor. The Pt functionalization was confirmed by TEM showing small Pt particles decorating the layers of WS_2_. HR-TEM images revealed interlayer spacings of 0.27 nm and 0.194 nm, which correspond to the (100) planes of WS_2_ and Pt, respectively. The sensor device was prepared by drop-casting of Pt-WS_2_ on Ti/Pt-based interdigitated electrodes and placed in a chamber with controlled humidity and temperature. The authors compared the sensor performance of pristine WS_2_ with Pt-WS_2_, observing that Pt-WS_2_ increased its humidity response up to 105.1X higher than pristine WS_2_. For pristine WS_2_ nanosheets, the sensitivity was determined to be 16.5 per RH%. However, with the introduction of Pt-decoration, the sensitivity significantly escalated to 1792 per RH%. In the following year, Gupta and collaborators [66] reported similar work, with the difference being that the humidity sensor was now based on Au-WS_2_. Au nanoparticles were well dispersed on the surface of WS_2_ with diameters ranging from 5 to 10 nm. In this case, Au-WS_2_ showed an enormous two orders of magnitude better humidity response than pristine WS_2_. The response of untreated WS_2_ devices ranged from 52 (at 25% relative humidity) to 1066 (at 75% relative humidity). In contrast, the response of the sensing device utilizing Au-functionalized WS_2_ nanosheets exhibited a broader range, spanning from 31 (at 25% RH) to 70,018 (at 75% RH). From these results, it can be highlighted that the type of metal used for the functionalization of TMDs has an extremely relevant effect on its electrochemical properties.

## 5. Raman Sensors

Raman spectroscopy is a versatile strategy to sense diverse analytes. The intensity of the weak Raman signals can be amplified by several orders of magnitude while reducing fluorescence in the analytes using surface-enhanced Raman scattering (SERS) platforms. Supported noble metal nanoparticles are commonly used for this purpose. The 2D-TMDs have been recently studied as SERS substrates as their electronic properties can be modulated to induce charge polarization, one of the mechanisms used to explain SERS. MoS_2_ has been used as a SERS substrate since charge transfer and dipole–dipole coupling occurs on the monolayer, processes responsible of Raman enhancement [67]. The 2D Janus MoSSe was used as a SERS substrate for detecting biomolecules. The Janus surface was prepared departing from MoSe_2_, followed by a sulfurization process to obtain MoSSe. TEM images allowed the authors to observe that the triangular structure of the single-crystalline MoSe_2_ monolayer sheets was maintained after the surface sulfurization. The Janus surface of MoSSe presented an out-of-plane dipole that polarized charges in biomolecules such as glucose and dopamine, enhancing their Raman signals up to 10^5^ [68]. N-doped, Ag nanoparticle decorated MoS_2_ and WS_2_ nanohybrids were used as fluorescence quenchers SERS substrates for the sensing of rhodamine B at concentrations as low as nM and later for the high sensitivity and reproducibility of polycyclic aromatic hydrocarbons such as pyrene, anthracene, and 2,3-dihydroxynaphthalene. The morphology of the decorated TMDs was observed by TEM microscopy, and the layered structure of MoS_2_ and WS_2_ was observed. Ag-nanoparticles in N-MoS_2_ had an average diameter of 3.4 nm and 2.2 nm for N-WS_2_. The Raman signals enhancement was attributed to charge transfer and dipole–dipole coupling [69]. Likewise, Singh and colleagues [70] reported the functionalization of MoS_2_ nanosheets with Ag nanoparticles using a hydrothermal process; in this case, the Ag-MoS_2_ sheets were used as SERS substrates for the detection of methylene blue. FESEM images showed the presence of spherical Ag nanoparticles with an average diameter of 35 nm on the surface of MoS_2_. This sensor presented an enhancement factor of 10^7^. The authors suggest that the combination of Ag and MoS_2_ facilitates enhanced charge transfer mechanisms, thereby improving the SERS sensing performance. Au-WS_2_ nanosheets were further functionalized with a specific aptamer to enhance the selectivity of a SERS sensor towards a cardiac marker myoglobin [71]. FESEM imaging showed the lamellar structures of WS_2_ with lateral sizes ranging from 50 to 100 nm. These structures were functionalized with Au-nanoparticles with a diameter average size of 29 nm. This biosensor was able to detect myoglobin in the 10 f mL^−1^ to 0.1 μg mL^−1^ concentration range.

**Figure 12:**
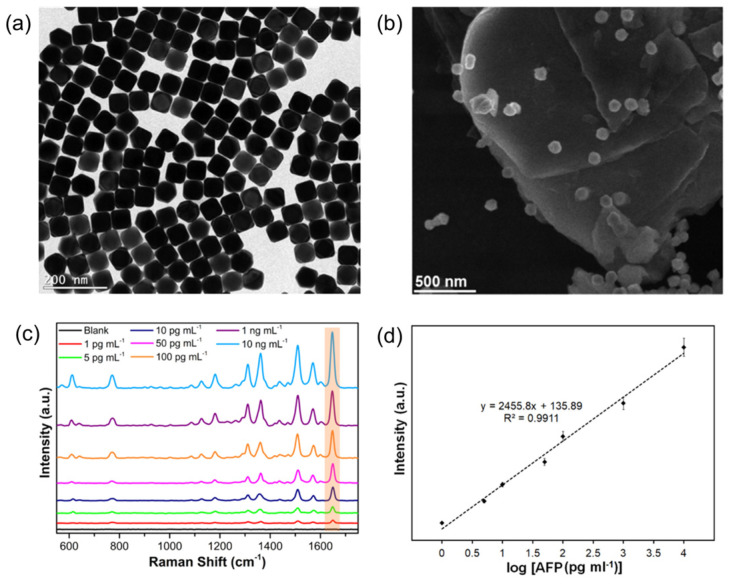
(**a**) TEM image of Au@Ag nanoparticles. (**b**) TEM image of Au@Ag-MoS_2_ substrates. (**c**) SERS spectra of R6G at various concentrations of AFP. (**d**) Calibration curve of the sensor response. Adapted from [72].

The identification of α-fetoprotein (AFP), a biomarker for hepatocellular carcinoma, was conducted utilizing MoS_2_ modified with an antibody targeting AFP and Au-Ag core-shell nanoparticles attached to a secondary antibody, thus establishing a sandwich-type SERS sensor [72]. The Au-Ag nanoparticles had a cubic morphology and an average diameter of 58 nm. SEM analysis of the SERS substrates showed the layered structure of MoS_2_ having micro-sized diameters and the cubic Au-Ag nanoparticles deposited on its surface. This platform was deposited on a silicon wafer and decorated with Ag-Au nanocubes to enhance the hot spots on the immunosensor (Figure 12). The newly designed SERS immunosensor demonstrated a broad linear detection span (ranging from 1 pg mL^−1^ to 10 ng mL^−1^) with an ultra-low limit of detection of 0.03 pg mL^−1^.

To amplify the chemical enhancement in SERS of molecules deposited on metal nanoparticles functionalized 2D-TMDs, Photo-Induced Enhanced Raman Spectroscopy (PIERS) has been used; this technique leverages electron migration from semiconductors to metal nanoparticles triggered by UV light exposure. Abid et al. [73] used PIERS to enhance the sensing performance toward 4-mercaptobenzoic acid of AuNPs-WS_2_. WS_2_ layers had an average lateral size of 100 nm, and the gold nanoparticles had an average radius of 27 nm. The photo-activation of WS_2_ results in a four-fold signal improvement compared to SERS from AuNPs-WS_2_ without UV irradiation.

**Figure 13:**
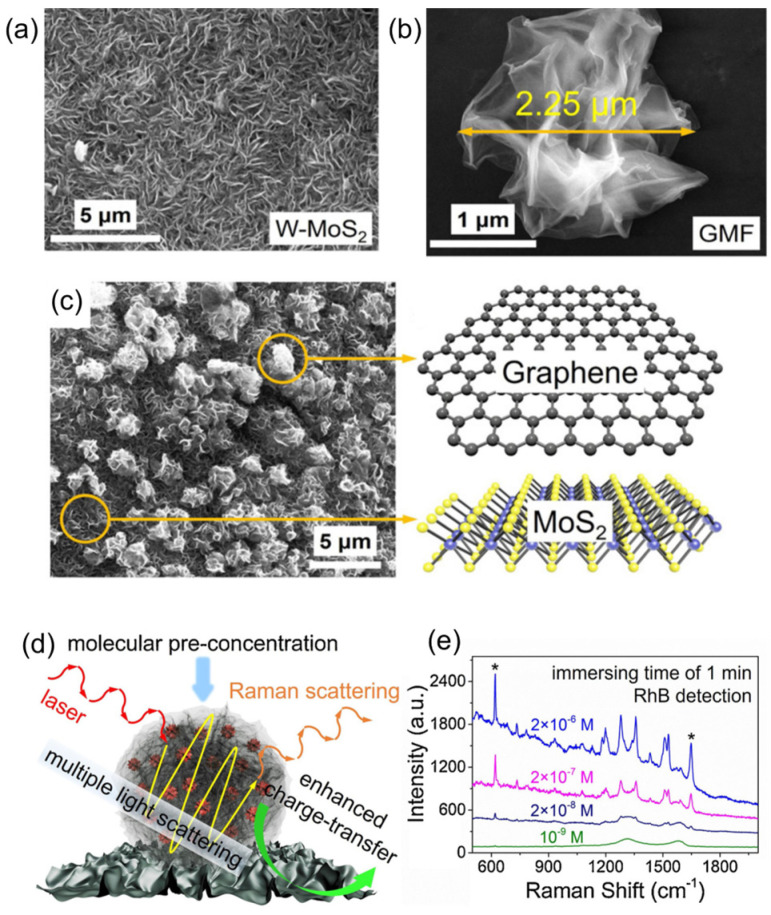
(**a**) SEM image of MoS_2_. (**b**) SEM image of GMF. (**c**) SEM image of GMFs/MoS_2_. (**d**) Schematic representation of SERS performance on GMFs/W-MoS_2_. (**e**) Raman spectral profiles of RhB across various concentration levels. * denotes the principal vibrational modes of RhB. Adapted from [74].

Besides metal nanoparticle functionalization, the 2D-TMDs functionalization with other constituents to improve their SERS performance was achieved. In 2020, MoS_2_ was decorated with graphene-microflowers (GMFs) [74]. The MoS_2_ platform had a flat surface with extensive internal corrugations, and individual GMFs had an average size of 2.25 μm. GMFs were effectively deposited onto the W-MoS_2_ platform. These GMFs acted as molecular enhancers, generating SERS ‘active regions’. GMFs/W-MoS_2_ showed an enhancement factor of 2.96 × 10^7^ for rhodamine B (Figure 13).

## 6. Concluding Remarks and Perspectives

The 2D-TMDs are semiconductors with a bandgap in the visible to n-IR frequencies in the electromagnetic spectrum. The almost full d-orbitals in the electronic structure of 2D-TMDs allow the layer-dependent bandgaps tuning, electrostatic coupling, and photo switching, making them excellent materials for field-effect transistors (FETs), ultrasensitive sensors, flexible electronics, fluorescence quenchers, and Raman enhancers [75]. In addition, 2D-TMDs can be easily integrated into membranes by a simple vacuum filtration methodology, improving the sensing mechanism in microfluidic and nanofluidic systems [76]. The electrical properties and the chemical structure of 2D-TMDs allow the design and production of adaptable transductors for electrochemical, optical, electrical, and SERS detection, demonstrating great potential for the massive production of flexible and reliable sensors. The 2D-TMDs produced by chemical methodologies such as CVD, hydrothermal, and liquid-phase exfoliation present defects, vacancies, and dangling bonds facilitating chemical functionalization.

Functionalizing 2D-TMDs provides a versatile means to tune and regulate their surface properties, expanding their potential applications in chemical sensors. One area where surface functionalization of 2D-TMDs has a major impact is in increasing the detection sensibility by making them more selective. While 2D-TMDs functionalized with noble metal nanoparticles are the most widely nanohybrid material used for sensing, there is an increasing interest in exploring more versatile functionalization approaches, including functionalization with proteins, DNA, and polymers, which encompasses a broader spectrum of possibilities for tailoring the sensing properties of 2D-TMDs. However, the quest for increased sensory performance in 2D-TMDs requires further research aimed at identifying specific molecules for the functionalization of 2D-TMDs, depending on the device to be developed. These molecules, once attached to the surface of 2D-TMDs, offer the potential to improve the selectivity towards specific analytes. Addressing the selectivity challenge by strategically functionalizing 2D-TMDs with molecules able to perform selectivity could pave the way toward highly specialized chemical sensors.

Finally, a crucial step is to subject the manufactured sensors to rigorous testing in samples and conditions that replicate real-world conditions. For example, evaluating sensors in human serum or blood for biomolecule detection, in polluted water for heavy metal detection, and in outdoor air for gas sensors. This practical application-oriented approach not only validates sensor performance, but also highlights the potential impact of 2D-TMDs in addressing critical challenges such as selectivity, sensibility, and stability.
